# Anhedonia in the psychosis risk syndrome: associations with social impairment and basal orbitofrontal cortical activity

**DOI:** 10.1038/npjschz.2015.20

**Published:** 2015-07-15

**Authors:** Victoria L Cressman, Scott A Schobel, Sara Steinfeld, Shelly Ben-David, Judy L Thompson, Scott A Small, Holly Moore, Cheryl M Corcoran

**Affiliations:** 1 Department of Psychiatry, Columbia University College of Physicians and Surgeons, New York, NY, USA; 2 The New York State Psychiatric Institute, New York, NY, USA; 3 Department of Neurology, Columbia University College of Physicians and Surgeons, New York, NY, USA

## Abstract

**Background/Objectives:**

Anhedonia is associated with poor social function in schizophrenia. Here, we examined this association in individuals at clinical high risk (CHR) for schizophrenia and related psychotic disorders, taking into account social anxiety. We then explored correlations between anhedonia and basal metabolic activity in selected forebrain regions implicated in reward processing.

**Methods:**

In 62 CHR individuals and 37 healthy controls, we measured social adjustment (Social Adjustment Self-Report Scale), social and physical anhedonia (Chapman Revised Anhedonia Scales), and social anxiety (Social Anxiety Scale for Adolescents) in cross-section. In a subgroup of 25 CHR individuals for whom high-spatial-resolution basal-state functional magnetic resonance imaging data were available, we also assessed correlations of these socio-affective constructs with basal cerebral blood volume in orbitofrontal cortex and related regions involved in reward processing.

**Results:**

Relative to controls, CHR individuals reported social impairment, greater social and physical anhedonia, and more social anxiety, exhibiting impairments comparable to schizophrenia. Regression analyses showed that anhedonia predicted social impairment and correlated negatively with basal cerebral blood volume within the orbitofrontal cortex (all *P*’s<0.05).

**Conclusions:**

Anhedonia and social anxiety are prominent in CHR individuals. Trait-like anhedonia may be a core phenotype related to orbitofrontal cortical function that, independent of symptoms, predicts social impairment. These data provide a rationale for interventions that target anhedonia and related activity in orbitofrontal cortical circuits in CHR individuals.

## Introduction

Social impairment is prevalent in schizophrenia, accounting for much of its morbidity.^1^ Moreover, it does not respond to therapies, with only a small effect (0.2) from dopamine antagonist pharmacotherapy,^2^ and an unclear effect as yet from remediation strategies that target social cognitive deficits.^3^ Importantly, this social impairment predates the onset of psychosis,^4–6^ and is characteristic of clinical high-risk (CHR) individuals^7^ at levels commensurate with the established illness.^8–10^ Across the schizophrenia spectrum, social impairment correlates with negative and affective symptoms^11–16^ and cognitive deficits.^7,17^ To develop novel effective interventions for social impairment in schizophrenia and its risk states, its underlying psychological and neural mechanisms must be characterized.

Social anhedonia and social anxiety are distinct psychopathological conditions that can contribute to social impairment in schizophrenia.^13,14^ As previously posited,^12,14,18^ anhedonia is a deficit in the capacity to experience positive reinforcement, including pleasure normally derived from social interactions, and manifests as low social drive. Social anxiety, on the other hand, is an active aversion to social interaction. Both social anhedonia and social anxiety are observed in schizophrenia and related to its social impairment.^14,19–21^ Social anxiety is evident at the first episode of psychosis^22,23^ and in youths identified as being at risk.^24^ Moreover, there is convergent evidence that anhedonia, as assessed with Chapman Revised Anhedonia Scales,^25,26^ is an enduring phenotype, associated with schizotypal traits in non-clinical populations, and a robust predictor of schizophrenia-spectrum disorders and related social impairment, including among psychometric schizotypes.^11–16,27,28^ Despite the well-established connection with schizotypy, anhedonia has not been analyzed using the Chapman Revised Anhedonia Scales in CHR individuals. Further, there is as yet no study of social anxiety and anhedonia as potential co-determinants of social impairment in CHR individuals. Our first aim, therefore, was to evaluate the contribution of anhedonia to social impairment in CHR individuals while also considering social anxiety. We hypothesized that both constructs would predict social impairment, but that anhedonia would be the primary contributor.

Our second aim was to examine the hypothesis that anhedonia in CHR individuals is related to activity in the orbitofrontal corticostriatal pathways, circuits recruited during the experience of both imagined and real rewards.^29–31^ In a subset of our CHR cohort for which high-resolution basal-state functional magnetic resonance imaging data were available, we examined correlations of anhedonia and social anxiety with basal-state metabolic activity in the orbitofrontal cortex (OFC) and related nodes involved in reward processing and/or negative affect.^32–36^ For anhedonia, we specifically hypothesized that Chapman ratings would be associated with decreased basal metabolism in the OFC.

## Subjects and Methods

### Participants

Participants included 62 CHR individuals and 37 healthy controls similar in demographics, ages 15–28 years, who were recruited through referrals by clinicians and schools, mailings of brochures, and via the Internet. This sample size is sufficient for detecting effect sizes of medium to large effect. Patients were help-seeking individuals who met criteria for the “attenuated psychotic symptom syndrome” using the Structured Interview for Prodromal Syndromes/Scale of Prodromal Symptoms.^37^ The Structured Interview for Prodromal Syndromes/Scale of Prodromal Symptoms was also used to assess positive and negative symptoms, which aggregate as separate factors.^38^ Exclusion criteria for all participants included history of psychosis; serious risk of harm to self or others; major medical or neurological disorder; and intelligence quotient (IQ) <70 (by history or administration of the Wechsler Adult Intelligence Scale^39^). Additional exclusion criteria for healthy controls included meeting criteria for a prodromal syndrome; family history of psychosis; history of adoption; and any Axis I diagnosis in the preceding 2 years (excluding substance use-related diagnoses), as assessed with the Diagnostic Interview for Genetic Studies.^40^ Written informed consent was provided by participants aged 18 years and older. For participants younger than 18 years, both written assent by the participant and written informed consent by a parent were provided. This protocol was approved by the Institutional Review Board of the New York State Psychiatric Institute at Columbia University.

### Measures

Instruments were selected based on availability of normative data for late adolescence and young adulthood. Assessments were done by trained clinical raters with a master’s level of clinical research training and experience. All questions were read aloud to participants, who endorsed or rated items. Of note, item overlap among the scales was found to be minimal (e.g., <15%).

#### Social adjustment

The Social Adjustment Scale, Self-Report^41^ has been used previously to document poor social adjustment in CHR cohorts, and its correlation with affective and negative symptoms.^8,10^ It covers four areas of social adjustment over the preceding 2 weeks: performance at expected tasks, amount of friction with others, finer aspects of interpersonal relations, and inner feelings and satisfaction. Ratings were along a five-point scale, with higher scores indicating worse social adjustment. “Overall mean” was calculated using the accompanying scoring manual, with domains not included if not applicable (i.e., “marital” when unmarried). The Social Adjustment Scale, Self-Report has been used in both community and clinical samples in depression and schizophrenia, and yields results similar to those obtained by interview format.^41,42^

#### Anhedonia

The Chapman Revised Physical and Social Anhedonia Scales have been validated in college students^25,26^ and used in schizophrenia cohorts.^14,20,30,43^ Higher scores reflect greater anhedonia. Item examples from the Physical Anhedonia Scale include, “I have seldom cared to sing in the shower”; “I have always had a number of favorite foods.” Social Anhedonia items sample interpersonal situations: “I have often enjoyed long discussions with other people.” These constructs have historically been significantly correlated.^44,45^

#### Social anxiety

The Social Anxiety Scale for Adolescents^46^ has been validated in adolescent and young adult cohorts.^46–48^ Participants indicated on a five-point scale self-statement items about activity or social preferences. Calculated scores include total score and three subscale scores—fear of negative evaluation, social avoidance and distress experienced generally in the company of peers (SAD-gen), and social avoidance and distress specific to new situations or unfamiliar peers (SAD-new). Higher scores reflect greater social anxiety.

#### Data analysis

Univariate models with risk status and gender as factors, and age as covariate, were used to analyze group differences in social impairment, anhedonia and social anxiety, and their subscales, with the expectation that symptomatic CHR individuals would have worse social function and greater levels of anhedonia and social anxiety than healthy peers. Pearson correlation analyses were used to evaluate associations between the socio-affective constructs of anhedonia and social anxiety, and their relationship with demographics and social impairment. Scatterplots were examined for outliers and parametric distribution: there was no evidence of outliers, skew, or deviations from a normal distribution for behavioral or imaging data. Regression model (with risk and gender as factors, and age as covariate) were then used to test the main hypotheses that anhedonia and social anxiety are related to social impairment. We also examined the relationship of social impairment to potential confounding covariates, including symptoms, IQ (available for a subgroup using the WAIS^39^), and prescription of antipsychotic medications (Supplementary Information). Further analyses were done to characterize identified associations of social impairment with psychological constructs (Supplementary Information). No adjustments were made for multiple comparisons as these additional analyses examined potential confounds or were descriptive in nature. All tests were two sided and alpha was set at 0.05 for all hypotheses, with trends reported for 0.05<*P*<0.10.

### Functional brain imaging

Imaging data from a subset of 25 CHR individuals (20 male, 5 female (mean age 21.4 years, s.e.m. 0.70)) available from a previous study^49^ were used for these exploratory analyses. Measures of cerebral blood volume (CBV) were obtained, which allow for the study of subregions of the brain in the basal state at submillimeter resolution. Subjects were resting quietly during scanning, and no tasks were administered. Oblique coronal T1 weighted images were acquired on a 1.5-Tesla Intera Philips scanner (TR, 20 mg; TE, 6 mg; FA 25°; 53 coronal slices, 0.78×0.78×3 mm voxels, no inter-slice gap, FOV 200 foot-to-head, 158 right-to-left, 159 anterior-to-posterior, matrix size 192×96) perpendicular to the hippocampal long axis before and 4 min after intravenous administration of the contrast agent gadolinium (0.1 mmol/kg Multihance). Basal-state CBV maps were generated as previously described;^49^ briefly, the differences between post- and pre-contrast CBV in each gray matter voxel was normalized for the post–pre difference scores for the top four pixels overlying the superior sagittal sinus.

For the OFC, the primary region of interest (ROI), the ROI was defined in the slice in which the gyrus rectus was best visualized immediately prior to the anterior midline merging of the corpus callosum. The gyrus rectus was identified as the most medial and inferior gyrus of the prefrontal cortex; the medial orbital gyrus was identified as the gyrus immediately lateral to the gyrus rectus. In addition to the OFC, basal CBV was also assessed within the ventromedial prefrontal cortex, as well as striatal and basal forebrain regions anatomically linked to the OFC and/or ventromedial prefrontal cortex, including anterior caudate, nucleus accumbens, amygdala, and subpallidal extended amygdala (Supplementary Figure S1).

#### Data analysis

All tests were two sided and alpha was set at 0.05 for the hypothesized association of anhedonia with reduced metabolic activity in the OFC. We then explored the association of each psychological measure with basal-state metabolic activity using forward stepwise regression models; for these exploratory analyses, there were no adjustments for multiple comparisons. Specifically, we examined associations of anhedonia, social anxiety, and social impairment with basal metabolism in cortical and subcortical regions related to both reward processing and negative affect.

## Results

### Sample characteristics

No significant differences were seen between CHR individuals (*n*=62) and controls (*n*=37) in age, gender, ethnicity, country of birth, and IQ (Table 1); variance was similar between the two groups. As expected, CHR individuals differed in symptoms, engagement with work or school, and use of medications (Table 1). Increasing age was associated with less physical anhedonia (*r*=−0.23, *P*=0.03); no constructs differed by gender. Age and gender were included as a covariate and factor, respectively, in further analyses.

### Social adjustment

**Figure 1:**
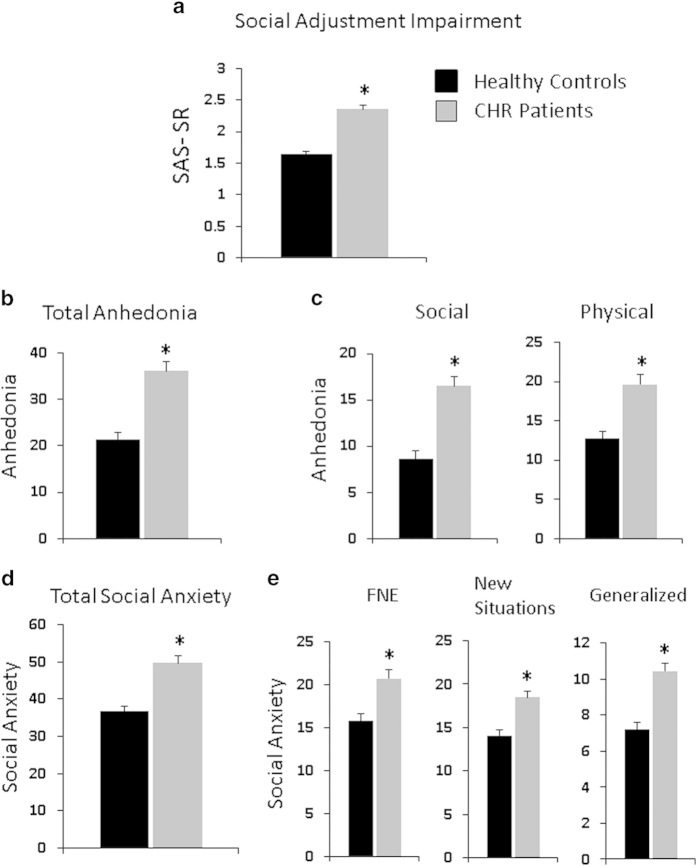
Mean scores for healthy controls (black bars) and clinical high-risk patients (CHR; gray bars) for measures of social adjustment impairment, anhedonia and social anxiety. Error bars represent the s.e.m. (**a**) SAS-SR (**b**) total scores for the Chapman Revised Anhedonia Scales (**c**) Chapman Social and Physical Anhedonia Scales. (**d**) SAS-A. (**e**) SAS-A subscales: FNE, social avoidance specific to new situations or unfamiliar peers (new situations), and social avoidance and distress in general (generalized). Higher scores indicate greater impairment on all scales. *Student’s *t*-test for group comparisons of CHR patients and healthy controls, *P*<0.01 (two tailed). CHR, clinical high risk; FNE, fear of negative evaluation; SAS-A, Social Anxiety Scale for Adolescents; SAS-SR, Social Adjustment Scale Self-Report.

Social adjustment was impaired in CHR individuals (F_3,96_=63.1, *P*<0.001) (Figure 1a); no gender or gender-by-risk interaction was evident.

### Anhedonia

Anhedonia was greater among CHR individuals, both as a summed score (F_3,96_=23.2, *P*<0.001; Figure 1b), and separately for social (F_3,96_=24.7, *P*<0.001) and physical (F_3,96_=12.6, *P*=0.001) anhedonia (Figure 1c), which were intercorrelated (*r*=0.59, *P*<0.001). Thus, we used total anhedonia (summed score) as the primary outcome, with additional separate analyses for social and physical anhedonia (Supplementary Information). There were no effects of gender or gender-by-risk interactions evident for any anhedonia analyses.

### Social anxiety

Social anxiety data were missing for one CHR individual and six healthy controls. Social anxiety was greater among CHR individuals, both for the total score (F_3,89_=15.8, *P*<0.001; Figure 1d) and for subscales (Figure 1e). Subscales were highly intercorrelated (CHR: all *r*’s>0.54, *P*’s<0.001; controls: all *r*’s>0.42, *P*’s<0.05, except for fear of negative evaluation with SAD-Gen (*r*=0.24, *P*=0.19)). Thus, total social anxiety was used in subsequent analyses. There were no effects of gender or gender-by-risk on social anxiety.

### Relationship with social impairment

**Figure 2:**
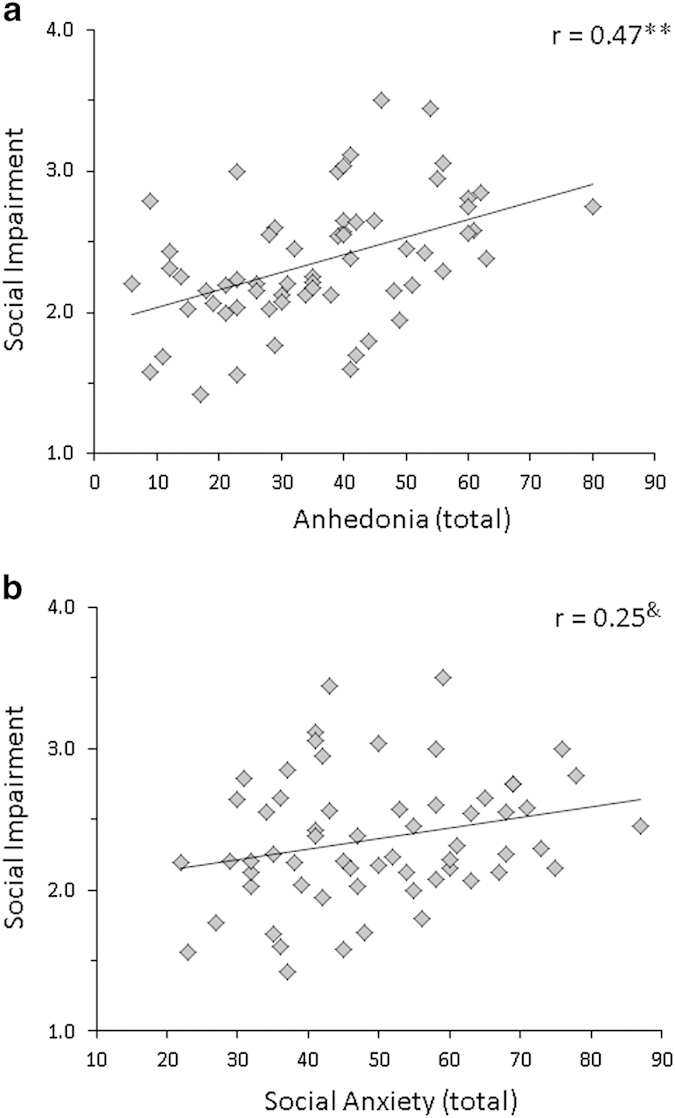
Scatterplots and correlations in clinical high-risk patients of social impairment with (**a**) total anhedonia and (**b**) total social anxiety. Pearson’s correlations; **P<0.001; Pearson’s correlation (trend) ^&^*P*=0.05.

Contributions of anhedonia and social anxiety to social impairment were examined together in a model that also included risk status, age, and gender. This model showed that social impairment was predicted by anhedonia and risk status, but not by social anxiety (Table 2a). In CHR individuals, only anhedonia predicted social impairment (Table 2b). In healthy controls, neither anhedonia nor social anxiety predicted social impairment (Table 2c). Scatterplots showed a significant correlation between social impairment and anhedonia (*r*=0.47, *P*<0.001; Figure 2a) and only a trend between social impairment and social anxiety (Figure 2b) in CHR individuals. However, the raw correlations between anhedonia (*r*=0.47) and social anxiety (*r*=0.25) with social impairment did not significantly differ (*P*=0.17). Additional analyses of the relationship between anhdonia and specific aspects of social impairment are shown in Supplementary Tables S1 and S2, respectively.

### IQ, symptoms and antipsychotic medications

With negative symptoms added, the model showed that both negative symptoms (*β*=0.37, *t*=3.31, *P*<0.01) and anhedonia (*β*=0.30, *t*=3.34, *P*<0.01) predicted social impairment. Neither IQ nor positive symptoms were related to anhedonia (summed, physical, or social), social anxiety, or social impairment (Supplementary Table S3). Removal of CHR individuals prescribed antipsychotic medications did not alter the predictive value of anhedonia for social impairment (Supplementary Table S4).

### Correlations with regional forebrain basal metabolic activity

**Figure 3:**
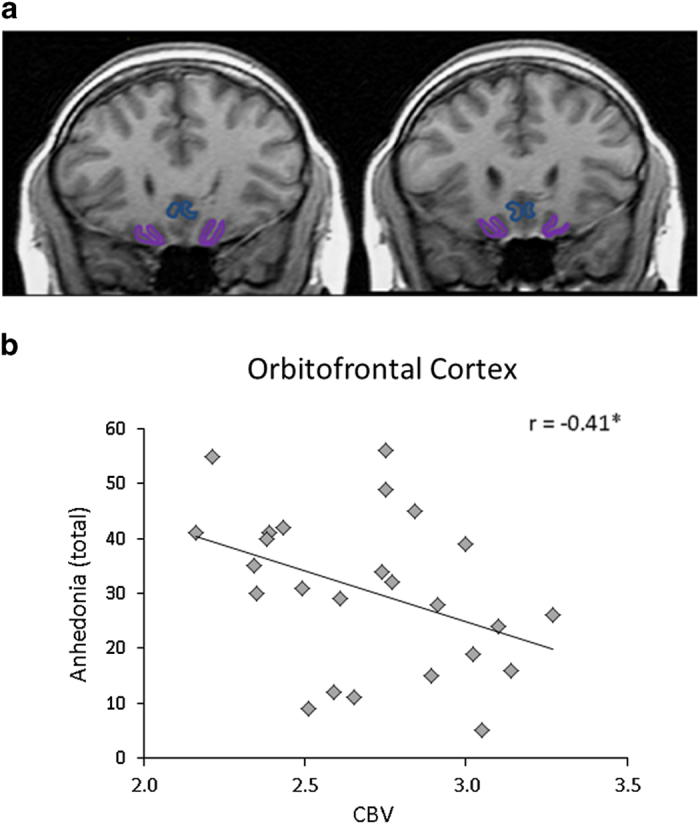
Correlation between anhedonia and basal cerebral blook volume in the orbitofrontal cortex. (**a**) Region of interest contours for the orbitofrontal cortex (in violet). (**b**) Correlation of resting CBV with total anhedonia. Pearson’s correlation; *P<0.05. CBV, cerebral blood volume.

Basal OFC activity was significantly and negatively correlated with anhedonia (*r*=−0.41, *P*<0.05; Figure 3); specifically physical anhedonia (*r*=−0.42, *P*<0.05; social anhedonia *r*=−0.24, *P*=0.24). This association between basal OFC activity and physical anhedonia remained strong when the analysis was confined to individuals not prescribed psychiatric medications (*r*=−0.43, *P*=0.06, *n*=18). Basal CBV values in the anterior caudate and nucleus accumbens were not associated with anhedonia; social anxiety did not correlate significantly with basal CBV in any of the brain regions examined (Supplementary Table S5).

## Discussion

### Main findings

High levels of anhedonia were prevalent in CHR individuals and predictive of marked social impairment, an association not accounted for by concurrent social anxiety, prodromal symptoms, intelligence, or medication use. Moreover, anhedonia, specifically physical anhedonia, was associated in CHR individuals with decreased basal activity in the OFC, a key node in a circuit important for reward processing and appetitive learning. This neural correlate of anhedonia was not explained by concurrent use of medications.

### Interpretation of findings in relation to previously published work

Social impairment is consistently observed in CHR individuals and related to both negative and affective symptoms.^7–10^ However, this is the first study to use Chapman scales in a CHR cohort to document profound anhedonia related to social impairment. Of note, the level of anhedonia in our risk cohort was comparable to or greater than that observed in cohorts of patients with established illness (CHR: social anhedonia=16.5; physical anhedonia=19.6; schizophrenia: social anhedonia=11.0–15.3; physical anhedonia=10.6–20.0).^13,14,16,20,25^ High levels of social anxiety^46^ were also prevalent in this CHR cohort, consistent with studies in schizophrenia^22,23^ and with a prior CHR study.^24^

The association between reduced OFC basal activity and Chapman anhedonia in a CHR cohort is a novel finding, but consistent with prior studies in schizophrenia.^30,50^ The OFC is normally recruited during the experience of both imagined and real rewards.^29,31^ It subserves valuation and effort-cost computations,^51^ which in schizophrenia are impaired and related to anhedonia and other negative symptoms.^52^ Prior studies in schizophrenia also implicate the striatum in abnormal reward processing (see refs 30, 50, 53, but see also ref. 54). We speculate that while striatal responses evoked by behavioral manipulations of motivation or positive-valence cues^30,50,53^ may relate to reward representation, low activity of striatal projection neurons under resting conditions may limit the utility of basal striatal CBV as a correlate for reward processing capacity or its converse, anhedonia. Finally, the finding of a trend relationship between anhedonia and lower basal activity in the subpallidal extended amygdala is in line with what is known about projections to the extended amygdala,^55^ and the responsiveness of the extended amygdala to both appetitive and aversive stimuli.^33,34^

Consistent with previous studies, we found that Chapman ratings of both social and physical anhedonia scales were correlated, and that total anhedonia is associated with social impairment. However, considering physical and social scales separately, we observed a significant correlation of physical—but not social—anhedonia with basal metabolism in the OFC. This finding is consistent with prior studies of the neural correlates of anhedonia.^30,56,57^ This may be because physical anhedonia items are relatively evocative—“dancing of flames in a fireplace”; “a brisk walk”; “smell of fresh bread”. Also, factor analyses show that the Chapman Social Anhedonia Scale has multiple small content clusters with modest associations, perhaps making it less amenable for correlational analyses with biological measures.^58^ Such potential scale differences, not relative social content, may account for greater sensitivity of the Physical Anhedonia scale to neural activity. All considered, however, the association of basal OFC activity with the Physical Anhedonia scale may reflect the primary hypohedonia described in (ref. 12) as a “pleasure impairment, a basic reduced capacity … [the basic source of which] is a deviation in the microanatomy or neurochemistry of the limbic system”.

### Strengths and limitations

The main strength of this study is its evaluation of the socio-affective construct of anhedonia, both in terms of real-life function and underlying neural circuitry, in a well-characterized cohort of youths at real and imminent risk for psychosis, who are already symptomatic yet who have limited exposure to dopamine antagonists, which themselves can lead to secondary negative symptoms and modification of reward circuits through dopamine receptor antagonism. Limitations include reliance on self-report scales, and modest statistical power for analyses of relationships with regional basal metabolic activity. In additional, since imaging data were not available for the control subjects, it is not clear whether the anhedonia–OFC basal metabolism relationship exists in the context of abnormal metabolic activity in CHR individuals.

### Implications for future research, policy, and practice

These findings have implications for treatment of social impairment in schizophrenia and its risk states, which are among the top 10 causes of disability worldwide.^59^ Social impairment is a predictor of schizophrenia outcome in high-risk cohorts,^60^ and persists beyond the first episode of psychosis in schizophrenia.^61^ As yet, there are no evidence-based treatments for social impairment (reviewed in ref. 10). The identification of the socio-affective construct of anhedonia as a main contributor to social impairment during the putative prodromal period, and the characterization of its neural correlates, can lead to new therapeutic approaches to address disturbances in motivation and emotional experience in schizophrenia and its risk states, thereby reducing morbidity.

Anhedonia may be a primary deficit in persons at risk for schizophrenia. Cognitive neuroscience paradigms of motivation and hedonics can be used to understand its underlying circuits in early stages of schizophrenia. These include the monetary incentive delay paradigm,^36^ and assessments of effort-based decision making,^52,62^ each of which activate orbitofrontal corticostriatal circuits. Using the monetary incentive delay in schizophrenia, decreased activation in frontostriatal regions in response to rewards (as compared with losses) is observed that correlates with negative symptoms.^54^ As for effort-based decision making, naturally occurring variation in dopamine activity in frontostriatal pathways is related to cost/benefit decision making in healthy individuals.^62^ Implementation of such paradigms in CHR cohorts in the context of functional imaging would allow assessment of what is likely a core phenotype in schizophrenia prior to the emergence of psychosis and not confounded by exposure to antipsychotic medications.

## Conclusions

Anhedonia is prominent and predictive of social impairment in the schizophrenia prodrome, and related to lower basal metabolic activity in the OFC. Future studies should determine how the trait-like neurobehavioral phenotype of anhedonia and related orbitofrontal activity relates to the capacity for reward processing, motivation, and ultimately functional outcome. Such studies will be useful in revealing novel neural-circuit targets for pro-motivational therapies in young people with emergent schizophrenia-related psychopathology.

## Figures and Tables

**Table 1:** Characteristics of the sample

	*Healthy controls*	*CHR individuals*
Total *N*	37	62
		
*Age*		
Mean (s.e.m.)	21.6 (0.5)	20.9 (0.4)
		
*Gender N (%)*		
Male	23 (62)	47 (76)
Female	14 (38)	15 (24)
		
*Ethnicity N (%)*		
Caucasian	19 (51)	28 (45)
African-American	9 (24)	13 (21)
Asian-American	3 (8)	6 (10)
Native American	1 (3)	0 (0)
More than one race	5 (14)	15 (24)
		
*Country of birth N (%)*		
USA	31 (84)	53 (86)
Other	6 (16)	9 (15)
		
*Employment/education N (%)* a		
Full-time work or school	12 (32)	5 (8)
Part-time work or school (<30 h)	20 (54)	37 (60)
Out of work or unemployed	5 (14)	20 (32)
		
*Symptoms (mean (s.e.m.))*		
SOPS total positive^a^	0.8 (0.2)	12.8 (0.6)
SOPS total negative^a^	1.0 (0.3)	13.9 (0.9)
		
*IQ*		
*N*_IQ_	24	38
Mean (s.e.m.)	111.4 (2.5)	112.9 (2.6)
		
*Medications N (%)*		
Antipsychotics^a^	0 (0)	8 (12.9)
Antidepressants^a^	0 (0)	15 (24.2)

Abbreviations: CHR, clinical high risk; IQ, intelligence quotient; SOPS, Scale of Prodromal Symptom.

a *P*<0.05.

**Table 2:** Regression models of anhedonia and social anxiety as predictors of social adjustment impairment

*Predictor*	*Standardized coefficient (*β)	*Student’s* t*-test*	*Significance (*P)
*a. Full cohort (F*_*5,87*_*=13.8,* P*<0.001)*			
Risk status	0.42	4.64	<0.001
Total anhedonia	0.35	3.88	<0.001
Total social anxiety	0.13	1.48	0.14
Age	−0.04	−0.55	0.59
Gender	−0.001	−0.10	0.99
			
*b. CHR individuals (F*_*4,57*_*=3.17,* P*=0.02)*			
Total anhedonia	0.43	3.56	0.001
Total social anxiety	0.13	1.06	0.29
Age	0.004	0.03	0.98
Gender	0.02	0.17	0.89
			
*c. Healthy controls—total anhedonia and social anxiety as predictors (F*_*4,26*_*=1.53,* P*=0.22)*			
Total anhedonia	0.17	0.94	0.36
Total social anxiety	0.25	1.33	0.20
Age	−0.28	−1.56	0.13
Gender	−0.20	−1.02	0.32
