# Supplementary material for: Anhedonia in the psychosis risk syndrome: associations with social impairment and basal orbitofrontal cortical activity
Source: NPJ Schizophr. 2015 Jul 15;1:15020–. doi: 10.1038/npjschz.2015.20 (PMC4849450; doi:10.1038/npjschz.2015.20)
Supplement: Supplementary Information [file npjschz201520-s1.doc]

**Supplemental Material**

**Supplemental Methods**

**Functional Brain Imaging** **Regions of Interest**

*Orbitofrontal and ventromedial cingulate cortices.* The orbitofrontal cortex region of interest (ROI) was completed in the slice in which the gyrus rectus was best visualized immediately prior to the anterior midline merging of the corpus callosum. First, the gyrus rectus was identified as the most medial and inferior gyrus of the prefrontal cortex and the medial orbital gyrus was selected as the gyrus immediately lateral to the gyrus rectus. In this same slice, the ventromedial (subgenual) cingulate ROI was included as the medial-wall gray matter gyrus immediately superior to the gyrus rectus and continued posteriorly for three slices terminating prior to the emergence of the septum.

*Anteromedial Striatum.* Within the anteromedial striatum ROIs for the anterior caudate and nucleus accumbens were defined. The ROI for the anterior caudate was defined in the coronal plane at its first unambiguous bilateral appearance and followed three to five slices until merging with the body of the structure was evident. The nucleus accumbens ROI was defined as immediately superior and lateral to the underlying septum and immediately inferior and lateral to the white matter of the internal capsule.

*Subpallidal Extended Amygdala:* Data on the amygdala proper were not available in all subjects. However, we were able to obtain data from the subpallidal extended amygdala, defined as gray matter beginning at the anterior dorso-lateral aspect of the amygdala, extending anteriorly and ventral to the anterior commissure to the ventro-posterior border of the nucleus accumbens .


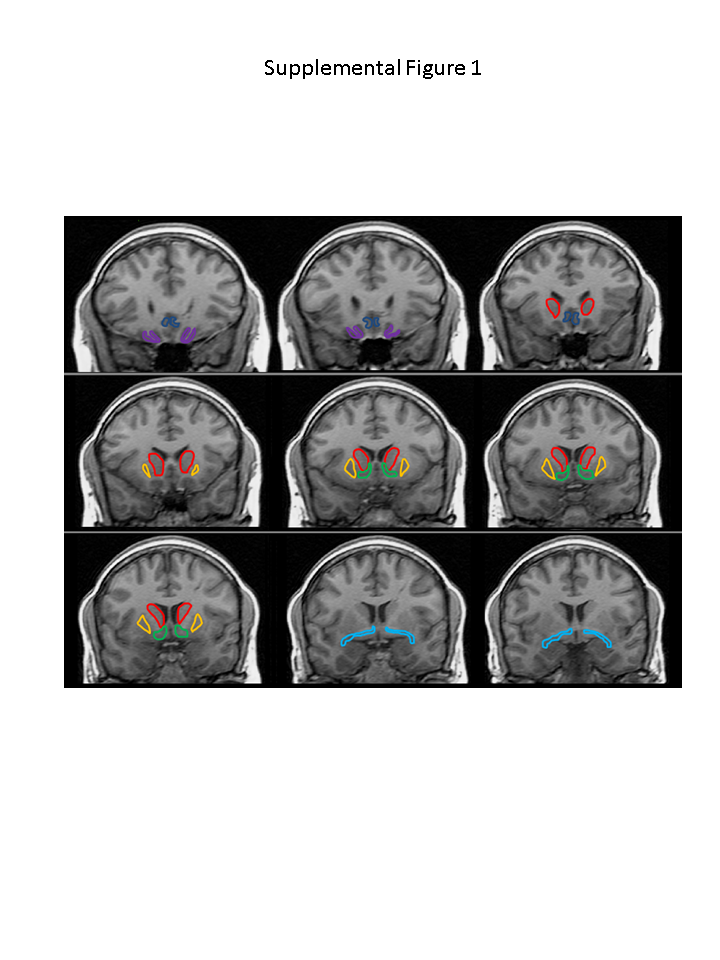


**Figure S1.** Region of interest contours for the brain regions in which CBV was quantified. Cortical regions: orbitofrontal (violet) and ventromedial cingulate cortex (dark blue) cortices; striatal and basal forebrain subregions: anterior caudate (red), nucleus accumbens (green), putamen (yellow; not used in this study) and the subpallidal extended amygdala (light blue).

**Supplemental Results**

**Table S1** Separate models for a) physical anhedonia and b) social anhedonia as predictors of social impairment, adjusted for social anxiety, age and gender.

| **Table S1a) PHYSICAL anhedonia as a predictor of social impairment** | | | |
| --- | --- | --- | --- |
| **Full cohort** (F_5,87_ = 13.8 , p < 0.001) | | | |
| Predictor | Standardized Coefficient (β) | Student t-test | Significance (p) |
| Risk status | 0.45 | 5.17 | **< 0.001** |
| Physical anhedonia | 0.31 | 3.65 | **< 0.001** |
| Total social anxiety | 0.14 | 1.59 | 0.12 |
| Age | - 0.02 | -0.23 | 0.82 |
| Gender | 0.02 | 0.25 | 0.81 |
|  | | | |
| **CHR individuals** (F_4,57_ = 3.17 , p = 0.02) | | | |
| Predictor | Standardized Coefficient (β) | Student t-test | Significance (p) |
| Physical anhedonia | 0.44 | 3.60 | **0.001** |
| Total social anxiety | 0.14 | 1.17 | 0.25 |
| Age | 0.06 | 0.46 | 0.65 |
| Gender | 0.04 | 0.33 | 0.74 |
|  | | | |
| **Healthy controls** (F_4,26_ = 1.53, p = .22) | | | |
| Predictor | Standardized Coefficient (β) | Student t-test | Significance (p ) |
| Physical anhedonia | 0.06 | 0.28 | 0.78 |
| Total social anxiety | 0.25 | 1.30 | 0.20 |
| Age | -0.30 | -1.64 | 0.11 |
| Gender | -0.22 | -1.04 | 0.31 |

| **Table S1b) SOCIAL anhedonia as a predictor of social impairment** | | | |
| --- | --- | --- | --- |
| **Full cohort** (F_5,87_ = 13.8 , p < 0.001) | | | |
| Predictor | Standardized Coefficient (β) | Student t-test | Significance (p) |
| Risk status | 0.44 | 4.66 | **< 0.001** |
| Social anhedonia | 0.26 | 2.96 | **.004** |
| Total social anxiety | 0.15 | 1.76 | 0.08 |
| Age | - 0.07 | -0.91 | 0.37 |
| Gender | - 0.03 | -0.35 | 0.73 |
|  | | | |
| **CHR individuals** (F_4,57_ = 3.17 , p = 0.02) | | | |
| Predictor | Standardized Coefficient (β) | Student t-test | Significance (p) |
| Social anhedonia | 0.31 | 2.44 | **0.02** |
| Total social anxiety | 0.18 | 1.38 | 0.17 |
| Age | -0.05 | -0.41 | 0.68 |
| Gender | 0.01 | 0.07 | 0.95 |
|  | | | |
| **Healthy controls** (F_4,26_ = 1.53, p = .22) | | | |
| Predictor | Standardized Coefficient (β) | Student t-test | Significance (p ) |
| Social anhedonia | 0.22 | 1.25 | 0.22 |
| Total social anxiety | 0.25 | 1.35 | 0.19 |
| Age | -0.28 | -1.56 | 0.13 |
| Gender | -0.23 | -1.22 | 0.23 |

**Table S2**. Anhedonia and social leisure impairment: Models of a) total, b) physical and c) social anhedonia as predictors of social leisure impairment, a subscale considered to be particularly informative for the age bracket of the CHR subjects.[^3^](#_ENREF_3)

| **Table S2a. Social leisure impairment** | | | |
| --- | --- | --- | --- |
| **Full cohort** (F_5,87_ = 14.5, p < 0.001) | | | |
| Predictor | Standardized Coefficient (β) | Student t-test | Significance (p) |
| Risk status | 0.36 | 3.76 | **< 0.001** |
| Total anhedonia | 0.37 | 4.02 | **< 0.001** |
| Total social anxiety | 0.10 | 1.14 | 0.27 |
| Age | - 0.05 | -0.56 | 0.58 |
| Gender | - 0.02 | -0.26 | 0.79 |
|  | | | |
| **CHR individuals** (F_4,57_ = 3.44 , p = 0.01) | | | |
| Predictor | Standardized Coefficient (β) | Student t-test | Significance (p) |
| Total anhedonia | 0.40 | 3.20 | 0.002 |
| Total social anxiety | 0.10 | 0.77 | 0.29 |
| Age | 0.004 | 0.03 | 0.44 |
| Gender | -0.05 | 0.41 | 0.68 |
|  | | | |
| **Healthy controls** (F_4,26_ = 2.49, p = .07) | | | |
| Predictor | Standardized Coefficient (β) | Student t-test | Significance (p ) |
| Total anhedonia | 0.47 | 2.70 | **0.01** |
| Total social anxiety | 0.19 | 1.04 | 0.31 |
| Age | -0.14 | -0.83 | 0.41 |
| Gender | 0.005 | 0.03 | 0.98 |

| **Table S2b. PHYSICAL anhedonia as a predictor of social LEISURE impairment** | | | |
| --- | --- | --- | --- |
| **Full cohort** (F_5,87_ = 13.2 , p < 0.001) | | | |
| Predictor | Standardized Coefficient (β) | Student t-test | Significance (p) |
| Risk status | 0.40 | 4.26 | **< 0.001** |
| Physical anhedonia | 0.32 | 3.45 | **0.001** |
| Total social anxiety | 0.12 | 1.23 | 0.20 |
| Age | 0.02 | -0.26 | 0.80 |
| Gender | - 0.004 | -0.05 | 0.96 |
|  | | | |
| **CHR individuals** (F_4,57_ = 2.81 , p = 0.03) | | | |
| Predictor | Standardized Coefficient (β) | Student t-test | Significance (p) |
| Physical anhedonia | 0.36 | 2.80 | **0.01** |
| Total social anxiety | 0.12 | 0.94 | 0.35 |
| Age | 0.01 | 0.10 | 0.92 |
| Gender | -0.04 | -0.27 | 0.79 |
|  | | | |
| **Healthy controls** (F_4,26_ = 1.69, p = .19) | | | |
| Predictor | Standardized Coefficient (β) | Student t-test | Significance (p ) |
| Physical anhedonia | 0.40 | 2.08 | **0.05** |
| Total social anxiety | 0.19 | 0.98 | 0.34 |
| Age | -0.16 | -0.90 | 0.38 |
| Gender | 0.05 | 0.23 | 0.82 |

| **Table S2c. SOCIAL anhedonia as a predictor of social LEISURE impairment** | | | |
| --- | --- | --- | --- |
| **Full cohort** (F_5,87_ = 13.1 , p < 0.001) | | | |
| Predictor | Standardized Coefficient (β) | Student t-test | Significance (p) |
| Risk status | 0.37 | 3.76 | **< 0.001** |
| Social anhedonia | 0.32 | 3.43 | **0.001** |
| Total social anxiety | 0.13 | 1.36 | 0.18 |
| Age | - 0.08 | -0.97 | 0.33 |
| Gender | - 0.05 | -0.61 | 0.54 |
|  | | | |
| **CHR individuals** (F_4,57_ = 2.66 , p = 0.04) | | | |
| Predictor | Standardized Coefficient (β) | Student t-test | Significance (p) |
| Social anhedonia | 0.35 | 2.70 | **0.009** |
| Total social anxiety | 0.13 | 0.99 | 0.33 |
| Age | -0.09 | -0.67 | 0.51 |
| Gender | -0.07 | -0.52 | 0.61 |
|  | | | |
| **Healthy controls** (F_4,26_ = 2.07, p = .12) | | | |
| Predictor | Standardized Coefficient (β) | Student t-test | Significance (p ) |
| Social anhedonia | 0.42 | 2.39 | **0.03** |
| Total social anxiety | 0.19 | 1.02 | 0.32 |
| Age | -0.16 | -0.90 | 0.38 |
| Gender | -0.08 | -0.45 | 0.66 |

**Table S3.** Correlations of IQ and positive symptoms with anhedonia, social anxiety, and social impairment in CHR individuals

|  | **IQ (N = 38)** | **SIPS positive symptoms (N =62)** |
| --- | --- | --- |
| Total anhedonia | -0.03 | 0.06 |
| Physical anhedonia | 0.02 | 0.05 |
| Social anhedonia | -0.08 | 0.06 |
| Social anxiety | -0.26 | 0.006 |
| Social impairment | -0.06 | -0.09 |

**Table S4.** Analysis of predictors of social impairment excluding CHR cases prescribed antipsychotic drugs (Controls: N=37; CHR: N=50)

| **Predictors** | | **Coefficients (β)** | **t** | **Sig.** |
| --- | --- | --- | --- | --- |
|  | Risk status | .373 | 3.877 | .000 |
|  | Total Anhedonia | .353 | 3.566 | .001 |
|  | Total Social Anxiety | .176 | 1.914 | .060 |
|  | Age | -.077 | -.948 | .346 |
|  | Gender | .040 | .481 | .632 |

**Table S5.** Spearman’s correlations between socioaffective constructs and brain metabolic activity

|  | **OFC** | **BA25** | **Anterior caudate** | **Ventral Striatum** | **Subpallidal Extended Amygdala** |
| --- | --- | --- | --- | --- | --- |
| **Total Anhedonia** | **-0.41***  (25) | 0.10  (25) | -0.18  (25) | -0.11  (25) | -0.36^&^  (25) |
| **Social Anxiety** | 0.12  (25) | -0.07  (25) | 0.14  (25) | -0.01  (25) | 0.25  (25) |

*p < = 0.05, ^&^p < 0.10

**References**

1. Britton, J.C.*, et al.* Neural correlates of social and nonsocial emotions: An fMRI study. *NeuroImage* **31**, 397-409 (2006).

2. Heimer, L. & Van Hoesen, G.W. The limbic lobe and its output channels: implications for emotional functions and adaptive behavior. *Neurosci Biobehav Rev* **30**, 126-147 (2006).

3. Barrantes-Vidal, N., Lewandowski, K.E. & Kwapil, T.R. Psychopathology, social adjustment and personality correlates of schizotypy clusters in a large nonclinical sample. *Schizophr Res* **122**, 219-225 (2010).
